# Determinants of Users’ Attitude and Intention to Intelligent Connected Vehicle Infotainment in the 5G-V2X Mobile Ecosystem

**DOI:** 10.3390/ijerph181910069

**Published:** 2021-09-25

**Authors:** Zhiyuan Yu, Doudou Jin

**Affiliations:** School of Journalism and Communication, Shandong University, Jinan 250100, China; jindd@mail.sdu.edu.cn

**Keywords:** 5G-V2X, ICV infotainment, technology acceptance model, user attitude and intention, mobile scenarios

## Abstract

With the accelerating industrialization of 5G-V2X and smart automobiles, the intelligent connected vehicle (ICV) integrated with sophisticated communication, caching, computing, and control techniques enhance the functionality of in-vehicle infotainment (IVI) and also provide more powerful telematic or entertainment choices in vehicular environment. The diverse needs of ICV users (e.g., drivers and passengers) can be satisfied during commuting and traveling. However, considering the limitations of transportation environment, the potential attitude and usage behavior for the upcoming ICV infotainment directly impacts on the traffic and road safety in sustainable cities. In this paper, we conduct an online and offline survey to investigate the key factors influencing the user attitude and intention of ICV infotainment, where the answers of a total of 502 valid respondents (i.e., IVI users) are collected in China. A conceptual technology acceptance model with the constructs of perceived usefulness (PU), perceived ease of use (PEOU), social influence (SI), consumer innovation (CI), and perceived risk (PR) is established, and then assessed via partial least square structural equation modeling. We find that the constructs of PU, PEOU, CI, and SI have a direct impact on attitude and usage intention, of which 46.8% and 73.4% of variance, respectively, are explained. The respondents show positive attitudes and higher usage intention towards the ICV infotainment. Although PR has insignificant path with attitude and intention, the driving experience moderation effect exists between PR and usage intention. We can see that ICV infotainment will become a trend in future transportation scenario. Through this survey, reference for traffic safety and usage norms will be provided to reduce the risky of public health issues (e.g., traffic accidents) in the context of ICV infotainment.

## 1. Introduction

With the advantages of massive connectivity, higher data rate, ultra-reliability, and lower latency empowered by 5G technique, 5G-V2X (vehicular-to-everything) as the new evolution of Internet of Vehicles (IoV) paves the way for next-generation Intelligent Transportation System (ITS) and automobile industry. Traffic safety and efficiency can be improved via perception, planning, and control in transportation environment [1,2]. Especially, the intelligent connected vehicle (ICV) equipped with the critical functions of communication, caching, computing, and control provides comfort usage experience and omnimedia services both for drivers and passengers in cabin environment, which elicits positive perceptions among consumers [3]. Altran predicts that the number of ICVs will be up to 360 million worldwide by 2022 [4], and the penetration of ICV with L2 and L3 level in China will reach 50% by 2025 [5]. Therefore, depending on onboard modules and road-side units, ICV gradually became a real-time information platform to undertake the role of decision-making, data discovery [6], and mobile content generation [7] in smart cities.

Compared with that of in-vehicle infotainment (IVI) in 3G/4G eras, ICV infotainment in 5G-V2X opens new opportunities not only for automakers and service providers, but also for the benefit of potential users in transportation scenarios. The driver and passengers can enjoy large amount of real-time, high-quality entertainment services by smoother interaction, such as instant congestion alters, vehicle-home connectivity, interactive holographic display, mobile conference, telemedicine, public surveillance, etc. The integrated solutions for ICV infotainment were developed using intelligent cockpit or IVI modules, e.g., long-range cruise, navigation, multiscreen interaction, head-up display, and AI-based voice assistant. Currently, although some drivers and passengers already develop the habit of infotainment services usage while commuting, it does not mean that ICV infotainment can be quickly recognized and receive widespread acceptance. There exists some obstacles and concerns in the early stages of 5G-V2X, for example, safety issues, operating friendliness, usefulness, and efficiency, etc. Besides, due to lack of understanding of 5G-V2X or ICV, there is also rejection and mistrust of ICV infotainment affected by social influence or individual factors (e.g., risk resistance, time or money cost). Therefore, it is necessary to study the technology acceptance of ICV infotainment and reveal the influencing factors among current IVI users. On this basis, the following research questions (RQs) are intended to address:

- RQ1: What is the relationship between user attitudes and behavioral intention for ICV infotainment;
- RQ2: How do the influence factors impact user attitudes and behavioral intention for ICV infotainment.

The rest of this paper is organized as follows: Section 2 illustrates the theoretical background of technology acceptance model and the related literature in ITS. Section 3 proposes the theoretical model and the associated hypotheses. Section 4 describes the demographic statistic results among respondents. Section 5 shows the analysis results of reliability, validity measurement, and hypothesis testing. Section 6 discusses the main findings, and then points out the limitations of the survey. Section 7 concludes this paper.

## 2. Literature Review

### 2.1. Theoretical Background

To improve the individual’s acceptance of new technology, researchers try to find the influencing factors and model those kinds of behaviors. A number of theoretical models of technology acceptance were proposed, such as Technology Acceptance Model (TAM), TAM2, and Unified Theory of Acceptance and Use of Technology (UTAUT).

Due to the explanation of the user’s behavior in the acceptance of information system [8] and the benefits of conciseness and empirical soundness [9], TAM was used in the fields of E-commerce, mobile payment, financial services, and autonomous driving, etc., where the explanatory and predictive power of individual usage behavior are recognized. TAM consists of perceived usefulness (PU) and perceived ease of use (PEOU) as important factors. The former refers to the degree of individuals who perceive that using a particular information technology improves their performance, while the latter refers to ease-of-use with the particular information system [10]. The specific usage behaviors are determined by their intentions, which are directly influenced by attitude and PU.

The modified TAM2 removed attitude and added the variables of subjective norm (SN), image, job relevance, result demonstrability (RD), output quality to influence PU, and two moderating variables (i.e., experience and voluntariness) [11]. TAM2 shows that SN influenced individuals’ behavioral intention (BI) when they used new technologies. Experience moderates the effect of SN on BI and PU. It means that individuals depend less on others’ opinions as usage experience increases. TAM2 further improves the explanation of TAM by introducing external variables that impact on internal beliefs (perceived usefulness).

UTAUT integrates a series of theories, e.g., theoretical diffusion theory, theory of planned behavior, motivational model, etc., which is seen as a new technology assessment tool that can not only understand the factors of technology acceptance, but also predict user behavior [12,13]. In contrast to the aforementioned model, UTAUT introduces age and gender as moderating variables, and takes into account both external variables and intrinsic motivation on BI and actual use behavior. However, the disadvantage is that it is potentially inadequate at explaining user acceptance of new technologies under voluntary conditions because the initial study only focused on large organizations where involuntary situations may exist [14,15].

Although the constructs are not new, the existing technology acceptance models were found to effectively measure the consumer’s behavior and successfully predict a large proportion of variance in users’ attitude and intention for the new technology.

### 2.2. Technology Acceptance Research in ITS

Driven by emerging techniques in next-generation ITS, especially for ICV, some prior works focus on the attitudes and BI of the telematics, phone-car connectivity and IVI systems, and autonomous driving, etc., as shown in Table 1.

- **Telematics**: Chen et al. found that attitude was the strongest factor influencing users’ intention to use telematics, and both PU and PEOU positively influenced attitude. In addition, perceived behavioral control also directly influenced BI [16]. Kim et al. demonstrated that PU and PEOU positively influenced users’ entertainment service satisfaction and information service satisfaction and design, respectively. Additionally, price affected users’ PU and intention [21].
- **Phone-car connected and IVI systems**: Park et al. confirmed the role of facilitating condition and technographics in positively influencing individual usage intention. However, being mobile-literate and having prior similar experience had no significant effect on intention, with mobile-literate referring to the trait of knowing how to use mobile media [17]. The study of user resistance of IVI in Korea revealed that former usage experience conduced negative influence on acceptance of IVI [18]. For voice interface of IVI, Kim et al. discovered the cost of switching influenced resistance to use voice-based IVI systems [22].
- **Autonomous driving**: Zhang et al. argued that PU, PEOU, perceived safety, and PR respectively influence individuals’ initial trust in L3 level autonomous vehicles, which determined individual’s attitude [20]. Liu et al. showed different acceptance for the level of autonomous and revealed that respondents perceived stronger benefits of fully autonomous vehicles than that of highly autonomous vehicles [23].

These functionalities or studies were initially developed in 3G/4G networks, which aim to improve the interaction of data flow and enrich IVI services [24]. Currently, aided by artificial intelligence, mobile communication, and computing, 5G-V2X empowered ICV is expected to support a wide range of applications for drivers and passengers. Meanwhile, the functionality of infotainment is deemed to be improved and diverse. More importantly, ICV infotainment, as an important part of smart city and smart transportation, effectively solves the problem of data island in in-vehicle infotainment in the 3G and 4G era, given that it is based on the advantage of data flow between the vehicle and the external environment. Thus, the vehicle can be turned into an in-car learning, leisure, and office location with ICV infotainment, or it can assist with city traffic management and security. Based our knowledge, there are few studies investigating technology acceptance of 5G-V2X-based infotainment in ICV. In this way, it is necessary to conduct a quantitative research to predict the usage attitudes and intentions among the users.

## 3. Research Model and Hypotheses

An adapted TAM is used to investigate the user attitudes and behaviors regarding ICV infotainment. In this paper, we design a structural equation model (SEM) integrating the constructs of consumer innovation (CI), social influence (SI), and perceived risk (PR) with perceived usefulness (PU), perceived ease of use (PEOU), attitudes (ATT), and behavioral intentions (BI) in TAM, which are defined in Table 2.

**Figure 1:**
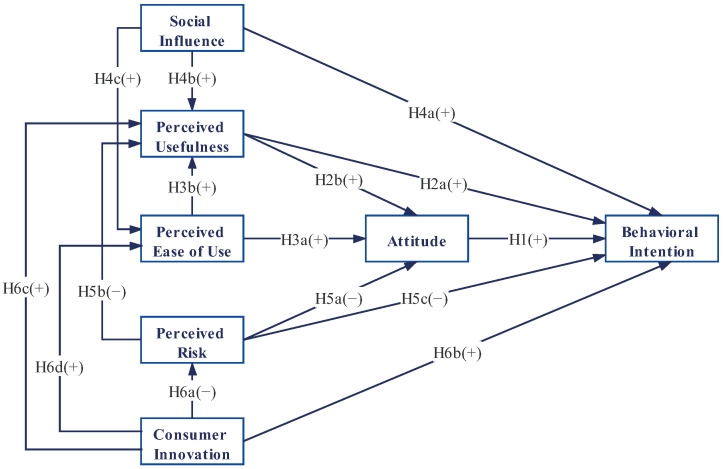
Proposed theoretical model.

According to the previous researches, we present our theoretical model in Figure 1, and then describe the causal relationship among the constructs as follows.

### 3.1. Attitude and Behavioral Intention

The successful acceptance of emerging technology depends on the users’ initial intention and continuous usage in everyday life [29]. However, the individuals’ attitudes towards information systems usually positively influence their behavioral intentions [28,30,31], which become one of the foundational variables in TAM. Vijayasarathy et al. found that consumers’ willingness to shop online was positively influenced by attitudes [28]. Van Dijk et al. found that positive attitudes directly influenced individuals to use the government internet services in Dutch [32]. Jonathan et al. confirmed that user’s attitude towards mobile banking positively influenced person’s intention [33]. Cao et al. proved that managers’ intentions to use AI for organizational decision-making was positively influenced by attitudes [34]. In summary, we recognize behavioral intention as the extent to which drivers and passengers are willing to accept ICV infotainment, and then engage in specific operating activities. We hypothesize:

**Hypothesis** **1** **(H1).** *Attitudes toward ICV infotainment have a positive influence on the BI to use.*

### 3.2. Perceived Usefulness and Perceived Ease-of-Use

Perceived usefulness and perceived ease-of-use are the key variables of TAM. Currently, in line with Davis’ findings [10], PU has a positive relationship with user attitudes and behavioral intentions. In other words, when consumers believe that new technology can improve productivity and bring convenience, they are more likely to use it [35]. For example, Schierz et al. found that mobile users’ PU for payment services had a significant positive effect on attitudes towards usage [9]. For the acceptance of shared parking services, Ning et al. demonstrated that PU had positive effect on both attitudes and BI, respectively. Moreover, PEOU had direct positive effect on PU [36]. Pengnate et al. confirmed that users’ PU of a website directly results in intention to use [37]. By integrating 88 empirical studies on TAM, King et al. revealed PU is a reliable factor with affecting user acceptance, which can be applied to a variety of study fields [38].

Unlike PU, the influencing role of PEOU in individual attitudes or behavioral intentions is controversial. Subramanian et al. found no significant effect of PEOU on individual adoption behavior of new technologies [39]. Hu et al. also confirmed in their study of physician use of telemedicine that PEOU did not have an effect on BI, which may reflect differences regarding the adaptation of TAM for various groups [40]. However, some scholars hold the opposite view. Agarwal et al. [41] and Lu et al. [42] found a significant positive correlation between PEOU and individuals’ intentions. Kasilingam et al. confirmed that PEOU positively influences attitudes for the chatbots, but showed no significance with intention [43]. We hypothesize as follows:

**Hypothesis** **2** **(H2a).** *Users’ PU of ICV infotainment positively influences BI.*

**Hypothesis** **2** **(H2b).** *Users’ PU of ICV infotainment positively influences attitudes.*

**Hypothesis** **3** **(H3a).** *Users’ PEOU of ICV infotainment positively influences attitudes.*

**Hypothesis** **3** **(H3b).** *Users’ PEOU of ICV infotainment positively influences PU.*

### 3.3. Social Influence

Social influence is the original subjective norm in the theory of planned behavior [44], which comes from peers and superiors, and is a determinant in behavioral intention [12,45]. The attitudes of user’s friends, colleagues, or family members towards the new technology may exert influence on themselves [46]. Especially in early usage stages, users have not yet established stable evaluation standards for the new technologies and are susceptible to the influence of social relations [47]. By feedback, the anxiety generated by the uncertainty of new technologies can be reduced [48]. Bhattacherjee et al. divided the sources of social influence into external and interpersonal influences, which come from mass media coverage or expert opinion, and friends & colleagues, respectively [49].

The positive impacts of SI on individual technology acceptance and usage behavior were demonstrated [41,48,50,51]. Fang et al. studied user acceptance of e-commerce and found that it had a direct positive influence on usage behavior [52]. Yang et al. revealed that SI positively influenced BI through the mediation of perceived dominance during the early stages for mobile payment services [53]. Leicht et al. confirmed a positive relationship between SI and consumer purchase intention for the self-driving [54]. In addition, some scholars found SI can be as an external factors to effect PU and PEOU. Lu et al. proved that SI positively influenced PU and PEOU in individuals’ acceptance of mobile internet services [42]. Sharif et al. revealed that SI had a favorable effect on both PU and PEOU for the usage of Internet technologies among South Asian educational institutions [55]. We hypothesize:

**Hypothesis** **4** **(H4a).** *SI positively affects individuals’ BI toward ICV infotainment.*

**Hypothesis** **4** **(H4b).** *SI positively affects PU of ICV infotainment.*

**Hypothesis** **4** **(H4c).** *SI positively affects PEOU of ICV infotainment.*

### 3.4. Perceived Risk

Perceived risk is a vital factor in influencing individual adoption or decision-making [56], which illustrates the uncertainty about the possible negative consequences due to the usage of emerging technology [26]. So, that perceived risk consists of many prospects in our society, e.g., cost risks, safety risks, and management pressures, etc. [57]. For example, consumers are aware of the perceived risk when they encounter possibly fake products online [58]. Except for the benefit of reducing accidents during autonomous driving, users are also concerned about the occurrence of equipment and system failure as major risks [59].

Featherman et al. found that perceived risk had a significant negative effect on perceived usefulness [26]. Lu et al. revealed that users’ PR of internet programs did not directly affect the usage intention, but it had a significant negative impact on individuals’ attitudes and PU [42]. Lee et al. found that attitudes towards using online banking were negatively affected by users’ PR of performance, time, finances, and security [60]. Crespo et al. revealed that PR did not affect perceived usefulness of the nonpurchaser group online, but had a significant negative effect on both attitudes and behavioral intentions. However, PR had a significant effect on the PU of the purchased group, but it did not affect attitudes [61]. We hypothesize as follows:

**Hypothesis** **5** **(H5a).** *Individuals’ PR has a negative effect on users’ attitudes towards ICV infotainment.*

**Hypothesis** **5** **(H5b).** *Individuals’ PR has a negative effect on the PU of ICV infotainment.*

**Hypothesis** **5** **(H5c).** *Individuals’ PR has a negative effect on BI of ICV infotainment.*

### 3.5. Consumer Innovation

Consumer innovation is a vital positive factor in new technology adoption behavior [54,62], which is referred to as individuals being eager to pursue something new and special with an openness to change [25,63]. As opposed to common consumers, innovative consumers can recognize the benefits of new products early on [64].

Van Raaij et al. showed that innovation positively influenced perceived ease-of-use for virtual education systems, but it had no significant effect on PU [25]. Schillewaert et al. found a positive effect of innovation on PEOU for automated sales systems, but the hypothesis on PU was not confirmed [65]. In low-risk situations, consumers had higher levels of innovation compared with that of high-risk situations. The PR negatively affected CI [66]. Aldás-Manzano et al. recognized the innovation is a prerequisite for PR because CI was revealed to negatively influence PR in the usage of online banking by consumers [67].

Moreover, the positive effect of individual innovation on user intention were confirmed [68,69]. Limayem et al. found that individual innovation directly results in users’ intention of online shop [70]. Bayus et al. demonstrated a positive relationship between individual innovation and new product adoption behavior [71]. In addition, the individual innovation can positively moderate attitudes and willingness of online searching and purchasing travel-related products [72]. We hypothesize that:

**Hypothesis** **6** **(H6a).** *CI negatively effects users’ PR towards ICV infotainment.*

**Hypothesis** **6** **(H6b).** *CI positively effects users’ BI towards ICV infotainment.*

**Hypothesis** **6** **(H6c).** *CI positively influences individuals’ PU of ICV infotainment.*

**Hypothesis** **6** **(H6d).** *CI positively influences individuals’ PEOU of ICV infotainment.*

## 4. Sample and Descriptive Analysis

We conduct questionnaire survey via the forms of offline and online from 23 June to 13 July 2021. For the local distribution, we aim to reach the respondents who were not active on the internet and the quality of samples can be controlled. For online collections, the link of the questionnaire was shared via social media, (e.g., WeChat, QQ, and Weibo) and survey platforms (e.g., WJX and wenjuan.com (accessed on 21 August 2021)), with the intention of collecting more geographically unrestricted samples. After completion of the survey, those respondents also shared the questionnaire, in which a snowball sampling method was used. We removed invalid questionnaires based on following criteria: (1) the respondents who never experienced the functions of IVI in era of 3G/4G and are completely ignorant of ICV; (2) including vacant values for particular item; (3) the completion time is less than 120 s; (4) all items are answered exactly the same. In this way, we try our best to make sure the participants have experience with IVI and have a certain knowledge about ICV. As a result, 502 of 798 valid questionnaires were collected. The effective recovery rate of this survey was 62.91%. For each measurement item presented in Table A1 of Appendix A, a seven-point Likert scale ranging from “Strongly Disagree = 1” to “Strongly Agree = 7” was adopted.

Table 3 shows the demographic characteristics by descriptive statistics. Of the valid respondents, 44.6% are male and 55.4% are female. For the age distribution, participants between 18–25 and 26–30-years-old are dominant, which account for 50.4% and 23.1%, respectively. In terms of education experience, 93.6% of respondents have a higher education. Therein, the majority of participants concentrates on the level of undergraduate (with 58.8%) and 21.3% of respondents experienced master or above education. For driving conditions, 85.46% of valid participants are drivers. Notably, 48.6% of respondents have at least 3 years driving experience. Based on the Decree of the Ministry of Public Security in China, the fresh driver can apply the driving license for urban bus, large-size trucks, and small-size cars, etc. Only with a driving experience of greater than 5 years can the large trucks and medium-size bus drivers obtain the qualification to drive large-size bus [73]. In this way, we categorize the driving experience into two groups: senior driver: over 5 years; novice driver: under 5 years.

We classify the income status of respondents into levels based on the National Bureau of Statistics of China ’s criteria for classifying low-, middle-, considerable-, and high-income groups [74]. To sum up, low-income respondents with a monthly income of less than 2000 CNY accounted for 28.5% of the total; middle-income respondents with a monthly income of 2000–5000 CNY accounted for 24.5%; 32.1% of respondents had considerable income of 5000–10,000 CNY, and high-income respondents of over 10,000 CNY accounted for 14.9%. Respondents from 29 of China’s 34 regions participated in the survey. Of these respondents, 19.3%, 10.4%, and 10.2% of respondents were from Shandong, Jiangsu, and Shanghai in eastern China, respectively; 10.2% were from Guangdong in southern China, and 7.6% were from the Ningxia Hui Autonomous Region in northwestern China.

## 5. Data Analysis and Results

Partial least square structural equation modeling (PLS-SEM) supports both exploratory and confirmatory investigations [75]. Notably, considering the goal of our study is theoretical prediction, PLS-SEM is a prediction-oriented tool and can be regarded as the preferred method [76] to evaluate both measurement and structural models [77]. Besides, it has less restriction in terms of measurement scale, sample size, and distribution [78]. In this paper, we use SmartPLS 3.2.9 to evaluate the proposed theoretical model. PLS algorithm is used to test the measurement model, which the maximum number of iterations and stopping criterion set as 300 and $10^{-7}$. For testing the structural model, PLS bootstrapping with settings of 5000 subsamples, bias-corrected and accelerated, and two-tailed hypotheses testing was conducted. Blindfolding keeps the default setting of omission distance at level 7 [79].

### 5.1. Reliability and Validity Measurement

In this study, internal consistency reliability and convergent and discriminant validity tests are used to evaluate the measurement model. The internal consistency reliability consists of Cronbach’s Alpha (α) and composite reliability (CR), for which the value of 0.70 and above is considered as acceptable value [80]. To assess convergent validity, indicator’ s outer loading (>0.70) and average variance extracted (AVE) (>0.50) are used to evaluate each construct [81,82]. Discriminant validity aims to make sure the construct differs from each other. Correlation between items in any two constructs should be lower than the square root of the average variance shared by items within a construct [83].

The CI1, CI2, and PR1 were canceled because of their loadings (0.595, 0.619, and 0.54) below the threshold 0.7. Table 4 shows the results of the reliability and convergent validity measures. We can see that the reliability of the measurement items is justified. Cronbach’ s Alpha (α) and CR range from 0.776 to 0.892 and 0.866 to 0.925, respectively, which indicate that the questionnaire has good internal consistency and high-reliability. The ranges of AVE values between 0.553 and 0.764 prove that all constructs passed the test and demonstrate good convergence validity. Table 5 shows Fornell–Larcker criterion result that the square root of AVE (in bold on the diagonal) is higher than the interconstruct correlations (off-diagonal values), in which discriminant validity was verified.

### 5.2. Hypothesis Testing

The completed results of the hypothesis testing for the structural model are presented in Table 6. Eleven of fifteen hypotheses are confirmed by the survey data.

For hypotheses H1, we can see that attitudes have a significantly positive correlation with behavioral intentions (β = 0.61; *p* < 0.001), which is the strongest among all hypotheses. As such, H1 is confirmed. Similarly, hypotheses H3a (β = 0.278; *p* < 0.001) and H3b (β = 0.427; *p* < 0.001) related to PEOU also prove to be strongly significant. This means that PEOU has a significantly positive effect on both PU and ATT. The results of H4a (β = 0.11; *p* < 0.01), H4b (β = 0.231; *p* < 0.001), and H4c (β = 0.363; *p* < 0.001) related to SI and H2a (β = 0.114; *p* < 0.05), H2b (β = 0.477; *p* < 0.001) related to PU are also supported after the hypothesis testing.

**Figure 2:**
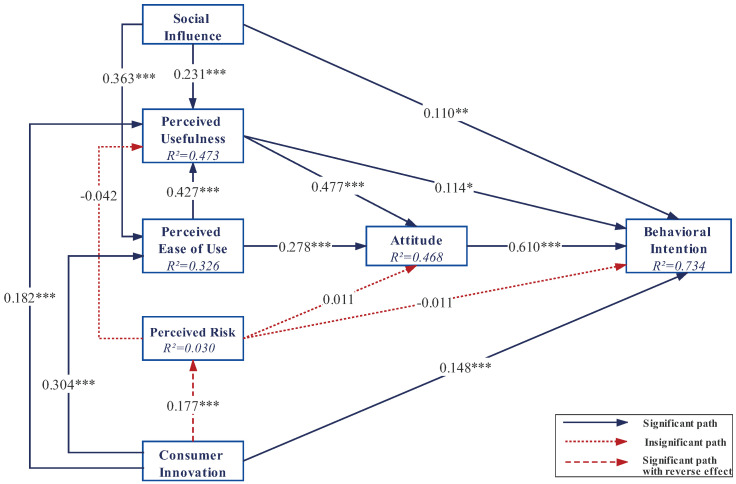
Structural model (*** *p* < 0.001, ** *p* < 0.01, * *p* < 0.05).

While all four hypotheses related to CI are tested with a significance (*p* < 0.000), the path coefficient of H6a is positive, contradicting our hypothesis that CI would have a negative effect on PR. Therefore, H6a (β = 0.177; *p* < 0.001) is not supported. H6b (β = 0.148; *p* < 0.001), H6c (β = 0.182; *p* < 0.001), and H6d (β = 0.304; *p* < 0.001) are confirmed. Besides, three hypotheses related to PR, i.e., H5a (β = 0.011, *p* = 0.755), H5b (β = −0.042, *p* = 0.367), and H5c (β = −0.011, *p* = 0.637) are not supported. The path coefficients and hypothesis testing are depicted in Figure 2.

The quality of PLS-SEM can be identified by $R^{2}$ values for the endogenous constructs with the thresholds of 0.75, 0.50, and 0.25 [81], which measure the strong, moderate, or weak predictive accuracy, respectively. $Q^{2}$ is used to evaluate the predictive performance. If its value is above zero, the latent constructs are explained to exhibit predictive relevance [76]. The value of $f^{2}$ is used to measure the effect size of a given construct, of which the thresholds of 0.02, 0.15, and 0.35 correspond to weak, medium, and strong effect sizes, respectively [77]. The values of $R^{2}$, $Q^{2}$, and $f^{2}$ are depicted in Table 7.

We use the value of adjusted $R^{2}$ as a criterion for judgment. The results show that 47.3% of the variance in PU is explained by CI, PEOU, PR, and SI. A total of 32.6% of the variance in PEOU is explained by SI and CI, while 46.8% of the variance in attitude is explained by PEOU, PU, and PR, and 73.4% of the variance in BI is explained by the antecedent variables in model. In terms of the amount of variance explaining, we can see that BI has a strong level of the predictive accuracy; ATT, PU, and PEOU are with relatively moderate level. In addition, the path model has predictive relevance for the endogenous structure because all $Q^{2}$ values are above zero. Finally, the effect sizes of the exogenous variables CI on PEOU, PR, PU, and BI are weak, which also applies to PEOU on ATT, PU on BI, SI on BI & PU, respectively. The effect sizes of PEOU on PU, PU on ATT, and SI on PEOU are all at medium-level. Only the effect size of ATT on BI is strong.

### 5.3. Moderation Analysis

Multigroup analysis (MGA) is regarded as an effective approach to analyze moderating effects in path models and is useful for discrete moderator variables [84]. As a nonparametric significance test, PLS-MGA enable to test whether differences between specific-group path coefficients are statistically significant, which does not rely on distributional assumptions [85]. To further investigate the hypotheses H5a, H5b, and H5c, following the same analysis approach in [43,86,87,88,89], MGA is used to determine whether the moderating effects exists or not from the aspects of respondents’ driving experience, knowledge about ICV, education, incomes, and gender. For each moderator, the samples are divided into two groups, in which the path coefficient and significant level are separately tested.

The analysis results are presented in Table 8. The path between PR on BI (H5c) is significantly different in the higher and lower driving experience groups. The effect of PR on BI (H5c) is moderated by driving experience with a significance level of 0.05 (Δβ=0.142, p=0.022<0.05), and it only has significance for respondents with higher driving experience (β=−0.127, p=0.012<0.05). It is statistically insignificant for the lower driving experience (β=0.015, p=0.582). Otherwise, the moderating effects of gender, education, income, and knowledge about ICV show no significance.

## 6. Discussion

This research identifies several key factors that influence individual acceptance of ICV infotainment, which includes attitudes, perceived usefulness, perceived ease of use, consumer innovation (CI), and social influence (SI).

In terms of attitudes towards ICV infotainment, the statistical results show that respondents give higher grade (Mean = 5.66, SD = 0.902) among four items on average, and 83.5% of respondents’ scores range from 5–7. Only 2.8% of respondents have relatively negative attitudes (score between 1 and 3), and 13.7% are neutral (score = 4). It means that potential users hold the positive attitudes regarding to ICV infotainment, which would make a solid foundation to develop the usage intention. Especially, the individuals’ attitudes towards ICV infotainment is the vital factor to influence their usage intentions (effect = 0.610). The stronger positive attitude is, the more likely individuals tend to use it, which answers RQ 1.

Both the PU (effect = 0.477) and PEOU (effect = 0.278) are important influential factors on attitudes, and PU directly affected BI. When participants find the functionalities of 5G-V2X-empowered infotainment are more useful and easier to use, their attitudes tend to be positive, with a high-willingness to use. In other words, the improvement of PU and PEOU can increase individual recognition and adoption for ICV infotainment. Otherwise, the nonadoption behaviors occur. For example, the ICV infotainment not only extends the connectivity and interoperability among the in-vehicles system, public spaces (e.g., workplace), and home with massive types of omnimedia form in smart cities, but also enhances the mobility services (e.g., accuracy positioning or road condition alerts, fatigue or obstacle detection via AI-aided computer vision) with higher data rate and less time delay. The drivers and passengers would like the benefits of ICV infotainment.

For PEOU, the precondition of ICV infotainment should guarantee driving safety and the second is user experience. If the ICV infotainment system is difficult to interact with or very costly in terms of time and spend, this results in negative attitudes. Moreover, PEOU is a strongest predictor (effect = 0.427) regarding to PU, which indicates that the easier the users found the ICV infotainment to use, the more likely the user tends to operate. The flexible interactions by different manners enhances the efficiency to access information. In 5G-V2X, the simpler interactive way can be realized. For example, supported by voice assistance, XR-HUD can project the instrument board and navigation data on front windows, which keep the drivers’ vision on the road and reduce the operating time according to user portrait database.

Our study also demonstrates that the CI and SI are two predictors for PU, PEOU, and BI. Compared with that of peers or common consumers, the innovators like to proactively acquire information and discover the benefits of ICV earlier [64]. The more innovative previous IVI users are, the better the PU and PEOU of ICV infotainment they have. This is because the innovators’ openness to novelty prompts their intentions to use ICV infotainment. Therefore, those innovators in high-probability turn to new users to enjoy ICV infotainment with spotlight features. Contrary to our hypothesis H6a, we found that individual’s innovation has a positive influence on PR regarding ICV infotainment. The reason can be explained that innovators are relatively active in accepting and experimenting the emerging technologies, and therefore, have a better grasp or clearer perception of the possible risks than the general consumer. The respondents believe they are able to judge whether they are at risk or not.

In the light of SI, the analysis results show SI has the positive effect on PU, PEOU, and BI among IVI users because individual experience and opinions are crucial to influence or give impressions to the potential customers and users through interpersonal communication or social media. In line with the study in [90], approximately 50% of messages about new vehicles for consumers are acquired in passive ways, and therein, more than half of passive information comes from friends or family members. The perceptions of ICV infotainment may be unstable during the developing progress and the potential user’s lack of first-hand usage experience. Through the awareness from acquaintances or other social media contacts who used to operate the functions in concept ICV model, they learn the advantages and estimate the degree of PU and PEOU. Finally, SI will determinate BI partly.

The perceived risk is related to the awareness of side-effect in usage (e.g., driving security and operation norms for vehicular and transportation environment), which is regarded as the major concern in automotive field. Different to prior works [42,60], we found that PR is not a significant predictor of PU, attitude, or BI. The reasons are as follows: firstly, the respondents used to experiencing the functionality of IVI are not sensitive to the time costs incurred in learning and setting up ICV infotainment. According to survey, the respondents’ overall attitude towards time spent on learning and setting up ICV infotainment is neutral (M = 4.03, SD = 1.723). Especially, 59.2% of respondents’ grade is equal or lesser than 4 points (40.4% for disagree and 18.8% for neutral). Secondly, compared with that of the know-nothing participants, the valid respondents with knowledge about ICV show a high level of understanding in emerging techniques, which may remove the perception of risk to some extent. Regarding the driving safety concern affected by Hacker controlling infotainment system, the overall evaluation is between neutral and comparatively agreeable (M = 4.80, SD = 1.530). As reported in [91], even if the IVI system is not operating, some drivers are also aware the PR for certain distracted behaviors, e.g., answering a phone call directly without connecting to the IVI system via Bluetooth. It implies that the effects of PR on ATT and BI towards the ICV infotainment may not be distinguished to some extent. In addition, we set the age, knowledge of ICV, gender, income, education, and driving experience as moderating variables to further analyze their respective moderating effects of PR on the PU, attitude, and BI. However, we found that driving experience is the only variable that can moderate PR and BI. The higher the awareness of PR for ICV infotainment, the less likely experienced drivers (greater than 5 years) are willing to use. As mentioned in [92], due to the distraction and optimism bias, more experienced drivers like to rely on their perceptions and are unwilling to accept the feedback from IVI system if contradiction occurs. In fact, senior drivers always place a higher value on driving safety. In summary, the mutual relationship between the proposed constructs are fully revealed and the RQ 2 is answered.

### 6.1. Implication

Based on the empirical analysis results, the theoretical and practical implication are summarized as follows.

For the theoretical implication, this study modifies the TAM by adding three factors, e.g., PR, CI, and SI. Although these three variables are relatively common, it is undeniable that present study provides certainly unique perspectives for future technology acceptance. First of all, there exists few studies about ICV infotainment in 5G era, and the results show that the IVI users value the usefulness and ease-of-use. Both the constructs originated from TAM still exert important influence on the acceptance of ICV infotainment. Secondly, perceived risk is a non-negligible factor in previous individual acceptance studies. In contrast, this study did not find direct influential effect on attitude and usage intention of ICV infotainment. Most respondents are not aware of the potential risks, which shows different sensitivities to risk regarding disparate research subjects. Thirdly, we found that the respondent’s driving experience is the key moderator rather than age or gender through moderating analysis. It would be interesting to consider the accumulated experiences of respondents involved in specific areas or systems when researching moderating effects. For example, in a study on the acceptance of new electronic products, similar prior experiences of respondents could be considered. Finally, as demonstrated in previous TAM extension studies, CI has a negative effect on PR. However, our study presents the opposite conclusion, which is that the positive impact between CI and PR needs to be considered.

In terms of practical implications, according to the empirical results, users hold a positive attitude and show higher usage intention towards ICV infotainment in 5G-V2X mobile scenarios. It means that the automakers and traffic management authority should think in advance and take measures to solve the potential issues. We need to actively guide the usage norms, and then make a trade-off between the traffic safety and user quality of physical experience based on the higher acceptance of upcoming ICV infotainment in 5G era. For example, according to the real-time traffic conditions and topography in urban and rural environment, ICV integrated with road-side infrastructures should dramatically allocate the vehicular (offline & local) and cloud (online & remote) resources to guarantee the mobile informative demands first. The addiction prevention system or setting is urgently deployed to rectify the abnormal operating behaviors in case of public health issues (e.g., traffic accidents).

### 6.2. Limitation

Firstly, the age of respondents mainly ranges from 18–40-years-old. The respondents above 40-years-old only account for 7.6%. Although automobile consumption market in China has a trend of getting younger, the post-90s generation is becoming the main demographic of automobile consumers. However, the aging of population cannot be ignored, and potential consumers from middle-aged group may show the huge purchasing power towards ICV. Secondly, the respondents are mainly from the developed provinces in terms of GDP, e.g., Shandong, Jiangsu, Shanghai in Eastern China, and Guangdong in Southern China, with less coverage of the less developed regions, e.g., Western China. Thirdly, since ICV infotainment is now in process of commercialization and industrialization, the majority of respondents only experienced LTE-V2X or simple functionality in telematics era. With the increase in users’ knowledge about ICV and more awareness of ICV infotainment, the perceptions are likely to change compared to that of the current situation. Therefore, a long-term observation with the control group is needed to further investigate ICV user attitudes and behavioral intentions.

## 7. Conclusions

In this paper, we have investigated the users’ (e.g., drivers and passengers) attitudes and behavioral intentions (BI) towards intelligent connected vehicle (ICV) infotainment in 5G-V2X scenarios among the participants who previously experienced in-vehicle infotainment (IVI) services. The influencing factors of perceived usefulness (PU), perceived ease of use (PEOU), consumer innovation (CI), social influence (SI), and perceived risk (PR) on users’ attitude and BI have been revealed. The proposed model explained 46.8% and 73.4% of variance for attitude and intention, respectively. Importantly, the users’ attitudes seriously affected usage behavior. Therefore, the variables of PU and PEOU exert huge influence on the attitude. We also found that IVI users opt to follow the advice from their friends and family members during the decision-making process. Innovative individuals are more receptive and find the benefits offered by ICV infotainment. As such, CI directly impacts BI. The indirect effect path between PR and BI has been found via the moderating group of senior driving experience. Compared with that of newly licensed drivers, the experienced drivers are aware of the driving risks of ICV infotainment, which conducted the negative effect of their BI.

## Figures and Tables

**Table 1:** Technology acceptance in Intelligent Transport System (ITS).

Subject and Source	Research Findings	Subject and Source	Research Findings
Automotive telematics [16]	Attitude→BI		PR→RS
	PEOU→Attitude		Resistance (RS) →IU
	PU→Attitude		TG→PU
	PEOU→PU		TG→PC
	Perceived Behavioural Control→BI		TG→PR
Smartphone-Car Connectivity [17]	Facilitating condition→Intent to use (IU)	IVI system [18]	PU→RS
	Technographics (TG)→IU		Perceived complexity (PC)→RS
Car navigation system [19]	PEOU→Attitude		SN→PU
	PU→Attitude		SN→PR
	PU→IU		Attitude→BI
	Attitude→IU		PU→BI
	Perceived locational accuracy (PLA)→PU		PEOU→PU
	Satisfaction (ST)→PU		PEOU→Attitude
	ST→PEOU	Automated vehicle [20]	Trust→Attitude
	Perceived system reliability (PSR)→ST		PU→Trust
	PSR→System & display quality (SDQ)		Perceived Safety Risk→Trust
	SDQ→Attitude		
	SDQ→IU		

**Table 2:** Construct definition and reference.

Construct	Definition	Reference
PEOU	The extent to which users perceives that it is easy to use ICV infotainment	Davis (1989) et al. [10]
PU	The extent to which users find ICV infotainment to be useful	Davis (1989) et al. [10]
CI	The ability of the users to tolerate change	Van Raaji & Schepers (2008) [25]
PR	The potential of loss in pursuit of desired outcomes when using ICV infotainment	Featherman & Pavlou (2003) [26]
SI	The extent to which users feel others recognize and encourage them to use ICV infotainment	Wu & Zhang (2014) [27]
ATT	The degree to which users perceive positive or negative feelings with ICV infotainment	Wu & Zhang (2014) [27]
BI	The users’ intention utilizes the functions of ICV infotainment	Vijayasarathy (2004) [28]

**Table 3:** Descriptive analysis.

Profile Category		Frequency	Percentage (%)
Gender (N = 502)	Male	224	44.6
	Female	278	55.4
Age	Under 18	2	0.4
	18–25	253	50.4
	26–30	116	23.1
	31–40	93	18.5
	41–50	35	7.0
	51–60	2	0.4
	Above 60	1	0.2
Education	Junior high school	8	1.6
	High school	24	4.8
	College	68	13.5
	Undergraduate	295	58.8
	Master’s and above	107	21.3
Driving experience	No driving license	73	14.54
	0 years (with driving license)	68	13.55
	1–2 years (including 1 year below)	117	23.31
	3–5 years	137	27.29
	6–10 years	72	14.34
	Over 10 years	35	6.97
Area	Northeastern China	27	5.4
	Eastern China	258	51.4
	Northern China	55	11.0
	the Central of China	29	5.8
	Southern China	57	11.4
	Southwestern China	25	5.0
	Northwestern China	51	10.2
Income (CNY per month)	Low income (Under 2000)	143	28.5
	Middle income (2000–5000)	123	24.5
	Considerable income (5000–10,000)	161	32.1
	High income (Above 10,000)	75	14.9

**Table 4:** Reliability and convergent validity.

Construct	Item	Loading	T Statistics	Cronbach’s Alpha	CR	AVE
ATT	ATT1	0.884	70.001	0.892	0.925	0.756
	ATT2	0.839	48.369			
	ATT3	0.879	68.810			
	ATT4	0.874	63.193			
BI	BI1	0.832	38.606	0.861	0.906	0.707
	BI2	0.831	47.923			
	BI3	0.885	84.982			
	BI4	0.813	41.293			
CI	CI3	0.861	57.715	0.845	0.906	0.764
	CI4	0.895	84.516			
	CI5	0.865	61.285			
PEOU	PEOU1	0.748	27.462	0.834	0.882	0.6
	PEOU2	0.746	29.788			
	PEOU3	0.796	46.021			
	PEOU4	0.805	41.954			
	PEOU5	0.775	32.647			
PR	PR2	0.811	8.419	0.776	0.866	0.684
	PR3	0.9	9.150			
	PR4	0.764	4.444			
PU	PU1	0.735	31.185	0.838	0.881	0.553
	PU2	0.774	32.983			
	PU3	0.806	42.023			
	PU4	0.721	28.571			
	PU5	0.717	25.852			
	PU6	0.703	23.308			
SI	SI1	0.837	46.289	0.825	0.883	0.655
	SI2	0.741	20.957			
	SI3	0.839	50.153			
	SI4	0.816	42.363			

**Table 5:** Discriminant validity (Fornell–Larcker criterion).

	ATT	BI	CI	PEOU	PR	PU	SI
ATT	**0.869**						
BI	0.834	**0.841**					
CI	0.485	0.549	**0.874**				
PEOU	0.578	0.551	0.476	**0.774**			
PR	0.038	0.063	0.177	0.024	**0.827**		
PU	0.652	0.641	0.488	0.63	0.043	**0.744**	
SI	0.712	0.673	0.475	0.507	0.182	0.526	**0.809**

**Table 6:** Path coefficients and hypothesis testing.

Hypothesis	Path	T Statistics	*p* Values	Path Coefficient	Bca (2.5; 97.5 )%	Result
H1	ATT → BI	12.119	0.000	0.61 ***	(0.505; 0.70)	Supported
H2a	PU → BI	2.513	0.012	0.114 *	(0.030; 0.201)	Supported
H2b	PU → ATT	8.649	0.000	0.477 ***	(0.358; 0.582)	Supported
H3a	PEOU → ATT	4.741	0.000	0.278 ***	(0.158; 0.395)	Supported
H3b	PEOU → PU	7.896	0.000	0.427 ***	(0.318; 0.529)	Supported
H4a	SI → BI	2.759	0.006	0.11 **	(0.034; 0.186)	Supported
H4b	SI → PU	4.383	0.000	0.231 ***	(0.127; 0.336)	Supported
H4c	SI → PEOU	7.162	0.000	0.363 ***	(0.257; 0.457)	Supported
H5a	PR → ATT	0.308	0.758	0.011	(−0.001; 0.072)	Not
H5b	PR → PU	0.883	0.377	−0.042	(−0.132; 0.049)	Not
H5c	PR → BI	0.471	0.638	−0.011	(−0.055; 0.034)	Not
H6a	CI → PR	3.632	0.000	0.177 ***	(0.014; 0.247)	Not
H6b	CI → BI	4.577	0.000	0.148 ***	(0.089; 0.215)	Supported
H6c	CI → PU	4.07	0.000	0.182 ***	(0.095; 0.26)	Supported
H6d	CI → PEOU	6.079	0.000	0.304 ***	(0.205; 0.403)	Supported

*** *p* < 0.001, ** *p* < 0.01, * *p* < 0.05.

**Table 7:** Values of $R^{2}$, $Q^{2}$, and $f^{2}$.

	Adjusted $R^{2}$	$Q^{2}$	$f^{2}$					
ATT	0.468	0.352	ATT→BI	0.53	PEOU→ATT	0.088	PU→ATT	0.259
BI	0.734	0.515	CI→BI	0.056	PEOU→PU	0.231	PU→BI	0.026
PEOU	0.326	0.193	CI→PEOU	0.106	PR→ATT	0.000	SI→BI	0.021
PR	0.030	0.015	CI→PR	0.032	PR→BI	0.000	SI→PEOU	0.152
PU	0.473	0.259	CI→PU	0.044	PR→PU	0.003	SI→PU	0.067

**Table 8:** Moderating effects analysis.

Path	Driving Experience		Absolute Value of Path Difference
	High (n = 107)	Low (n = 395)	
	Over 5 years	Under 5 years	
PR→BI	−0.127 *	0.015	0.142 *
PR→PU	−0.064	−0.008	0.056
PR→ATT	0.009	0.004	0.005
	**Knowledge level about ICV**		
	High (n = 125)	Low (n = 377)	
	More and Well	A little and Have some	
PR→BI	−0.078	0.015	0.093
PR→PU	−0.086	−0.013	0.073
PR→ATT	0.067	−0.019	0.086
	**Education**		
	High (n = 402)	Low (n = 100)	
	Bachelor’s degree or above	Below Bachelor’s degree	
PR→BI	0.011	−0.096	0.107
PR→PU	−0.068	0.11	0.178
PR→ATT	0.038	−0.081	0.119
	**Income**		
	Considerable and high (n = 236)	Low and middle (n = 266)	
	Over CNY 5000	Under CNY 5000	
PR→BI	−0.060	−0.010	0.050
PR→PU	0.027	−0.136	0.163
PR→ATT	0.022	−0.008	0.029
	**Gender**		
	Female (n = 278)	Male (n = 224)	
PR→BI	−0.001	−0.064 *	0.063
PR→PU	−0.086	−0.041	0.046
PR→ATT	0.033	0.055	0.022

* *p* < 0.05.

## Data Availability

Not applicable.

## Abbreviations

The following abbreviations are used in this manuscript:

ATT	Attitude
AVE	Average variance extracted
BI	Behavioural intentions
CI	Consumer innovation
CR	Composite reliability
ICV	Intelligent Connected Vehicle
IoV	Internet of Vehicles
ITS	Intelligent Transportation System
IVI	In-Vehicle Infotainment
PEOU	Perceived ease of use
PR	Perceived risk
PU	Perceived usefulness
SEM	Structural equation model
SI	Social influence
TAM	Technology Acceptance Model
UTAUT	Unified Theory of Acceptance and Use of Technology

## Appendix A. Questionnaire Item

**Table A1:** Constructs and measurement items in the questionnaire.

Construct	Item and Content	Source
Perceived ease of use(PEOU)	PEOU1: I would find functions of ICV infotainment easy to use	Davis (1989)
	PEOU2: I would find operating ICV infotainment is easy to learn	
	PEOU3: I think ICV infotainment functions easily do what I want	
	PEOU4: It is easy for me to be skillful at using ICV infotainment	
	PEOU5: I can flexibly interact with ICV infotainment system	
Perceived usefulness (PU)	PU1: ICV infotainment will help me spend driving time efficiently	Kim et al. (2016)
	PU2: I can obtain necessary information via ICV infotainment	
	PU3: Information provided by infotainment will be useful for me while driving	
	PU4: ICV infotainment let me kill time or relieve pressure for driving	Jiang et al. (2015)
	PU5: ICV infotainment will help me solve difficulties in driving **†**	Self-developed
	PU6: ICV infotainment functions will reduce my dependence on my phone while driving **†**	
Consumer Innovation (CI)	CI1: I have a positive attitude toward innovations **§**	Leicht et al. (2018)
	CI2: I am open-minded toward new products **§**	
	CI3: I know the names of new products before other people	Goldsmith & Hofacker (1991)
	CI4: In general, I am the first among friends to buy new products	
	CI5: I would be interested enough to buy new products if available	
Perceived Risk (PR)	PR1: I think ICV infotainment would cause privacy loss that personal information may be used without informed **§**	Featherman & Pavlou (2003)
	PR2: Hackers may take control infotainment system to impede driving safety	
	PR3: Setting and learning to use ICV infotainment may waste my time	
	PR4: I am concerned that ICV with infotainment is too expensive	Liu (2019)
Social Influence (SI)	SI1: My colleagues’ beliefs about ICV infotainment encourage me to use it	Wu & Zhang (2014)
	SI2: My colleagues’ thoughts influence the degree to which I use ICV infotainment	
	SI3: I would consider to use ICV infotainment if someone personally recommended it	Al-Somali et al. (2009)
	SI4: The media report about ICV will prompt me to try infotainment functions **†**	Self-developed
Attitude (ATT)	ATT1: Using ICV infotainment is good idea	Taylor & Todd (1995)
	ATT2: Using ICV infotainment is a wise idea	
	ATT3: I like to use the functions of ICV infotainment	
	ATT4: Using ICV infotainment functions would be pleasant	
Behavioral Intention (BI)	BI1: I will use ICV-based infotainment functions on a regular basis in the future	Moon J-W & Kim Y-G (2001)
	BI2: I will strongly recommend others to use ICV infotainment	
	BI3: I will frequently use ICV infotainment in the future	
	BI4: With same price, I prefer to buy a ICV with more infotainment functions **†**	Self-developed

**†**: Items PU5, PU6, SI4 and BI4 are self-developed; **§**: PR1, CI1 and CI2 were removed after evaluation because their outer loadings were less than the critical value of 0.7.
